# Knowledge mapping analysis of sedentary behavior and mental health research: A bibliometric analysis from 2004 to 2024

**DOI:** 10.1097/MD.0000000000044275

**Published:** 2025-09-12

**Authors:** Yi Liu, Zhen Zhang, Hao Zhang, Sisheng Tian

**Affiliations:** a Institute of Chinese Medical Literature and Culture, Shandong University of Traditional Chinese Medicine, Jinan, Shandong, China; b YangSheng College of Traditional Chinese Medicine, Guizhou University of Traditional Chinese Medicine, Guiyang, China; c Experimental Center, Shandong University of Traditional Chinese Medicine, Jinan, Shandong, China.

**Keywords:** Citespace, emotional disorders, mental health, sedentary behavior, visualization

## Abstract

**Background:**

Mental health issues exhibits a significant correlation with sedentary behavior (SB). To construct a scientific knowledge map of SB and mental health using bibliometric methods and to explore the research status, hotspots, and future trends in this field over the past 20 years.

**Methods:**

Articles were obtained in the Web of Science core collection database, with SB and mental health topics. Simultaneously, visual analysis of the included literature was cooperation among countries/institutions, core authors, active journals, and co-occurrence of keywords, and a scientific knowledge map was drawn.

**Results:**

From 2004 to 2024, publications on the topic of SB and mental health have shown a rapid growth trend, albeit with a slight decline after 2022. Regarding cooperative relationships, cooperation among countries, institutions, and authors is close, with the United States, Kings Coll London, among others, occupying core positions in this field. The International Journal of Environmental Research and Public Health is the most prolific journal. Current research hotspots include “association between sedentary behavior and physical activity with health,” “association of sedentary behavior with depression, anxiety, and risk factors” and “associative diseases such as obesity and cardiovascular mortality risk in children and the elderly.” Future research directions maybe “influence of sedentary behavior on sleep and specific psychological disorders.”

**Conclusion:**

This study underscores the significant attention given to SB and mental health over the past 2 decades, providing valuable insights and guiding future research endeavors.

## 1. Introduction

Sedentary behavior (SB) refers to any activity with an energy expenditure lower than 1.5 metabolic equivalents in a state of wakefulness, such as watching TV, using a computer, sitting in transit, leaning, or lying down, occurring in various settings, including work, study, and leisure environments. Data shows that the majority of awake adults spend most of their time sitting,^[1]^ with Chinese individuals spending an average of 160 minutes per day sitting during leisure time.^[2]^ Particularly after the outbreak of the coronavirus disease 2019 (COVID-19) pandemic in China, people’s mobility and activity intensity decreased, leading to an increase in sedentary time. In the post-pandemic era today, this situation has not significantly improved with the acceleration of mechanization, automation, and informatization processes. As a relatively new research area, SB has attracted increasing attention from scholars, and more and more studies have confirmed the impact of SB on health. Research shows that even with recommended levels of physical activity (PA), prolonged SB each day can still have adverse health effects, such as causing individuals to develop myopia, decreased bone density, and sleep disorders,^[3]^ and even leading to obesity, hypertension, cardiovascular diseases,^[4,5]^ cancer,^[6]^ and various metabolic diseases,^[7–9]^ becoming a major public health issue.

Furthermore, the relationship between mental health and SB has been a focus of attention.^[10,11]^ A systematic review suggests that SB exacerbates mental health issues in children and adolescents, leading to anxiety, depression, and other psychological disorders.^[12]^ Additionally, an analysis of data from the Global School-based Student Health Survey indicates a significant correlation between SB and anxiety and depression symptoms in both boys and girls. For instance, adolescents who spend >2 hours sitting each day have a higher risk of anxiety and depression symptoms compared to those who sit for <2 hours per day.^[13]^ Data from the 2021 Asia’s Best Workplaces (Mainland China Region) survey reveals a high detection rate of anxiety tendencies among working professionals, with severe SB and lack of physical exercise being identified as independent risk factors for anxiety tendencies.^[14]^ The impact of SB on mental health has become a scientific issue of great concern. However, the research progress in this field lacks clear and coherent analysis.

Bibliometrics, a scientific method gradually gaining prominence in recent years, is widely used for dynamic analysis of development trends in the medical field.^[15]^ CiteSpace (Drexel University, Philadelphia ), developed in the Java programming environment, is a visualization tool for bibliometric analysis.^[16]^ This software can analyze literature data in a specific research field and present it in the form of a graph, allowing for a clear understanding of the development trends and research hotspots in that field, thereby providing references for research directions in the field.^[17]^

## 2. Materials and methods

### 2.1. Data sources and retrieval strategies

Using the Web of Science (WOS) core collection as the database source, we conducted a literature search on January 8, 2024. The search strategy, revised based on published literature,^[18–20]^ is as follows: TS = (“sedentary behavior” OR “sedentary behavior” OR “sedentary time”) AND TS = (([mental OR mood psychological OR psychology] AND [health OR illness OR disease* OR disorder* OR wellbeing OR well-being]) OR depress* OR anxiety). We set the time limit from January 8, 2004, to January 8, 2024. The document types included were “Article” or “Review Article,” with the language restricted to English. A total of 3092 publications were retrieved.

### 2.2. Data filtering

**Figure 1.**
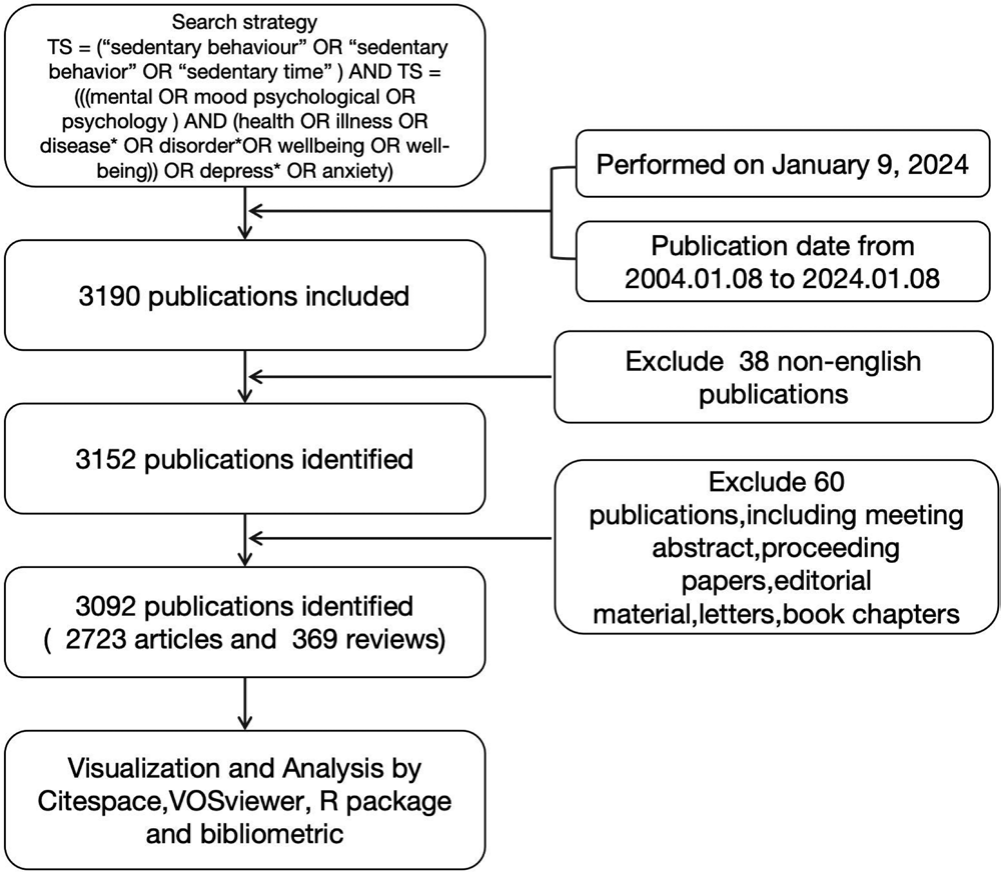
Flowchart of literature selection.

Inclusion criteria: literature that meets the search strategy. Exclusion criteria: duplicate publications; conference notices, achievements, popular science, etc; literature with incomplete information such as publication time, authors, institutions, keywords, etc, as outlined in Figure 1 for publication screening and inclusion process. During the literature evaluation process, 2 researchers independently screened the literature. When disagreements occurred: first, the 2 researchers conducted in-depth discussions on the controversial literature, carefully compared and analyzed the reasons for their different views, and referred to relevant guidelines and criteria in the field. If the differences still could not be resolved through discussion, a third senior researcher in the field was invited to participate. The final decision on whether to include the literature was made through joint discussion among the 3 of them.

### 2.3. Data processing and analysis

Citespace software is utilized for literature data analysis with the following parameter settings: time slicing focuses on literature from 2004 to 2024, with each year serving as a slice; the threshold for TOP N in each slice is set to 50, and the *k* value for the g-index is set to 25. For node types, including author, resource, institution, country, and keyword, the threshold is set to top N per slice = 25. Pathfinder and pruning networks graph trimming algorithms refine the graph for clarity. Betweenness centrality indicates the importance of nodes in a network (in addition to degree centrality, closeness centrality, etc). CiteSpace utilizes this metric to discover and measure the significance of literature, highlighting it with purple circles to signify its importance. Nodes with high betweenness centrality often serve as “turning points” in connecting different clusters, as frequently mentioned in CiteSpace.

## 3. Results

### 3.1. General information

**Figure 2.**
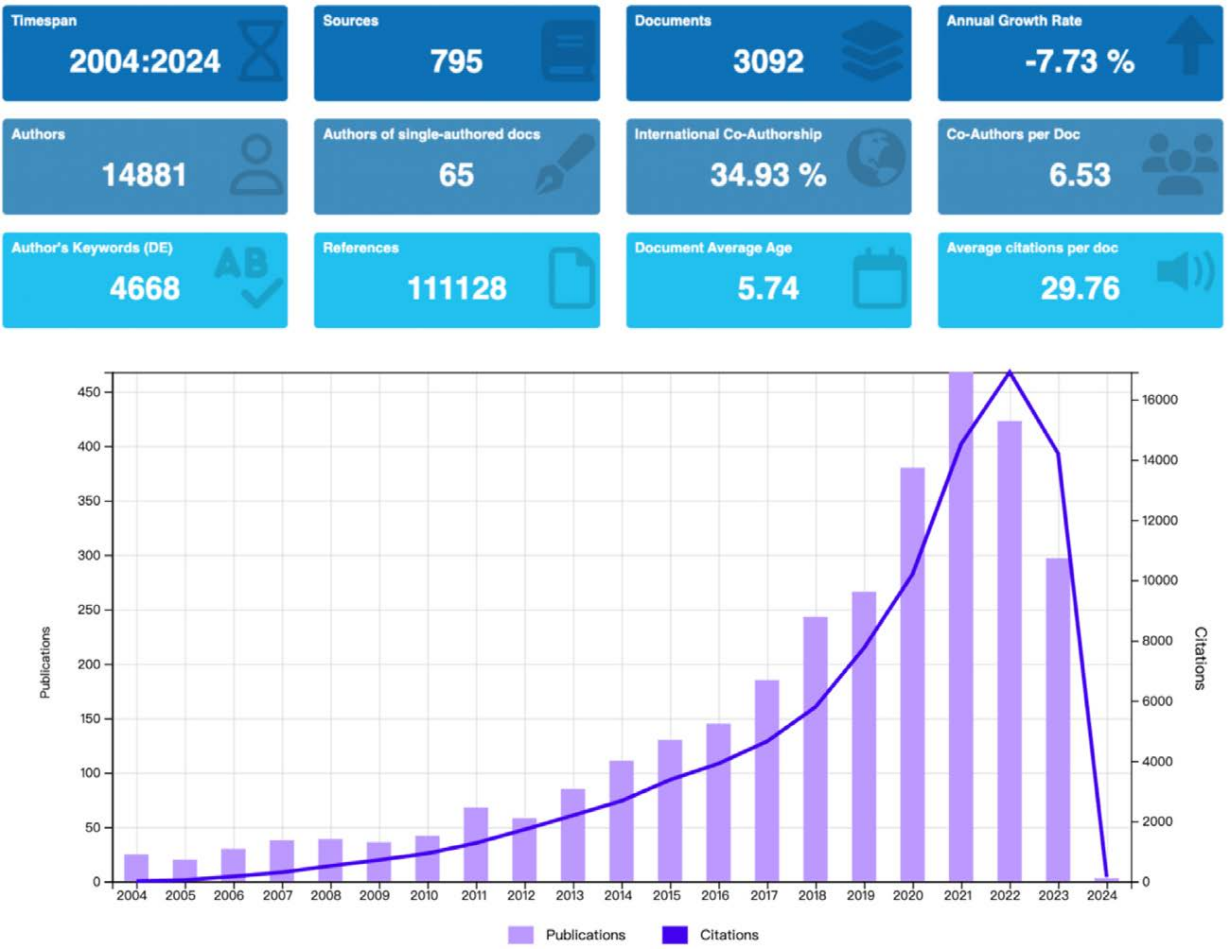
Basic information and annual number of publications over 2004 to 2024.

We conducted a literature search on “sedentary behavior and mental health” in the literature indexed in WOS from 2004 to 2024. Ultimately, 3092 publications were included, involving 102 countries/regions, 606 institutions, 14,881 authors, and 795 journals (see Fig. 2).

### 3.2. Analysis of annual publication

Between 2004 and 2024, 3092 publications were produced on “sedentary behavior and mental health.” We observed that the publication output experienced a slow growth period from 2005 to 2017, followed by a rapid increase from 2018 to 2021 and a decline in activity from 2022 to 2023, indicating that related research is expected to stabilize. Specifically, there were 24 publications in 2004, which slightly decreased to 20 publications in 2005. From then until 2021, the annual publication output continued to rise, reaching 468 publications. From 2021 to 2023, the annual publication output gradually decreased but remained 297 publications. Meanwhile, the annual citation count for research on this topic consistently increased until 2022, highlighting its research significance (see Fig. 2).

### 3.3. Research country/regional analysis

**Figure 3.**
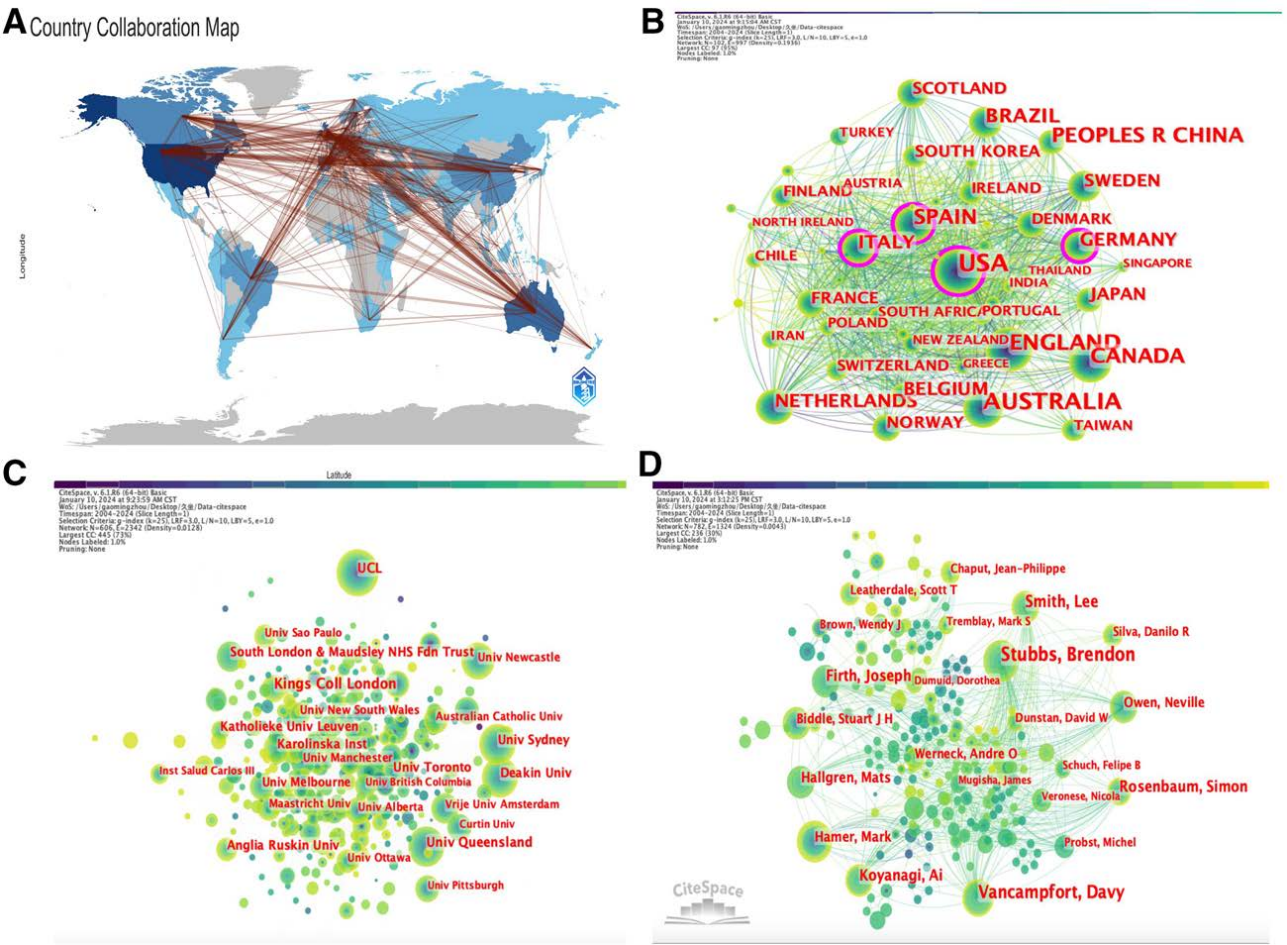
The visualization of countries/regions, institutions and authors. (A) Country collaboration map. (B) Visualization map of countries. (C) Visualization map of institutions. (D) Visualization map of authors.

Research in this field involves 102 countries/regions. The highest-producing country is the USA (n = 817), followed by Australia (n = 486), England (n = 467), Canada (n = 312), and the People’s Republic of China (n = 252). Among the top 10 highest producing countries, the USA has the highest centrality at 0.18, followed by Germany (centrality = 0.16), Italy (centrality = 0.13), England (centrality = 0.1), and Spain (centrality = 0.1), as shown in Table 1. Moreover, close global cooperation among countries forms a stable cooperative network, as depicted in Figure 3A, B.

**Table 1:** Top 10 countries/regions ranked by publication number and centrality.

Rank	Countries	Count	Countries	Centrality
1	USA	817	USA	0.18
2	Australia	486	Germany	0.16
3	England	467	Italy	0.13
4	Canada	312	England	0.1
5	People’s Republic of China	252	Spain	0.1
6	Spain	251	India	0.08
7	Brazil	200	Netherlands	0.05
8	Netherlands	164	France	0.05
9	Germany	138	Denmark	0.05
10	Belgium	133	South Africa	0.05

### 3.4. Analysis of research institutions

Research in this field involves 606 institutions. The highest-producing institution is Kings College London, contributing 111 publications to this field, followed by University College London (UCL, n = 80), University of Queensland (n = 79), Deakin University (n = 73), and Karolinska Institute (n = 72). Among the top 10 highest-producing institutions, UCL has the highest centrality at 0.09, and the same centrality is observed for Karolinska Institute, indicating that these institutions hold a central position in research cooperation, as shown in Figure 3C and Table 2.

**Table 2:** Top 10 institutions ranked by publication number and centrality.

Rank	Institutions	Count	Institutions	Centrality
1	Kings Coll London	111	UCL	0.09
2	UCL	80	Karolinska Inst	0.09
3	Univ Queensland	79	Univ Queensland	0.07
4	Deakin Univ	73	Univ Sydney	0.07
5	Karolinska Inst	72	Harvard Med Sch	0.07
6	Univ Sydney	69	Deakin Univ	0.06
7	South London & Maudsley NHS Fdn Trust	68	Univ Melbourne	0.06
8	Katholieke Univ Leuven	63	Univ Toronto	0.05
9	Anglia Ruskin Univ	61	Univ Ghent	0.05
10	Univ Toronto	61	Columbia Univ	0.05

UCL = University College London.

### 3.5. Analysis of research authors

Research in this field involves 14,881 authors. The most prolific author is Brendon Stubbs, contributing 96 publications, followed by Vancampfort D (n = 76), Smith L (n = 49), Koyanagi A (n = 45), Firth J (n = 41), Rosenbaum S (n = 40), Hallgren M (n = 36), Hamer M (n = 29), Owen N (n = 26), and Werneck AO (n = 26). In terms of citation count, Vancampfort D has the highest citation frequency (284), followed by Tremblay Ms (274), World Health Organization (266), and Stubbs B (213), with citation frequencies exceeding 200. In terms of influence, Stubbs B has the highest h-index (h_index = 31), followed by Vancampfort D (29), Firth J (23), Hallgren M (20), and Rosenbaum S (20), as shown in Figure 3D and Table 3.

**Table 3:** Top 10 productive authors, co-cited author and influential authors.

Rank	Authors	Record count	% of 3092	Co-cited author	Count	Element	h_index
1	Stubbs B	96	3.105	Vancampfort D	284	Stubbs B	31
2	Vancampfort D	76	2.458	Tremblay MS	274	Vancampfort D	29
3	Smith L	49	1.585	World Health Organization	266	Firth J	23
4	Koyanagi A	45	1.455	Stubbs B	213	Hallgren M	20
5	Firth J	41	1.326	Ekelund U	189	Rosenbaum S	20
6	Rosenbaum S	40	1.294	Schuch FB	185	Smith L	19
7	Hallgren M	36	1.164	Teychenne M	142	Koyanagi A	18
8	Hamer M	29	0.938	Biddle SJH	128	Owen N	17
9	Owen N	26	0.841	Hamer M	127	Biddle SJH	16
10	Werneck AO	26	0.841	Firth J	125	Hamer M	16

### 3.6. Analysis of published journals

Research in this field involves 795 journals. The most prolific journal is the *International Journal of Environmental Research and Public Health* (n = 226), followed by *BMC Public Health* (n = 160) and *PLOS ONE* (n = 108), all of which have published over 100 articles. In terms of citation count, the most cited journal in *Medicine & Science in Sports & Exercise* (n = 1617), followed by *PLOS ONE* (n = 1450), *International Journal of Behavioral Nutrition and Physical Activity* (n = 1249), *Preventive Medicine* (n = 1193), *BMC Public Health* (n = 1172), *Lancet* (n = 1133), and *American Journal of Preventive Medicine* (n = 1044). These journals have citation frequencies exceeding 1000, as shown in Table 4.

**Table 4:** Top 10 productive journals and cited journals.

Rank	Journal	Count	% of 3092	Cited Journal	Count
1	*International Journal of Environmental Research and Public Health*	226	7.309	*Med Sci Sport Exer*	1617
2	*BMC Public Health*	160	5.175	*PLOS ONE*	1450
3	*PLOS ONE*	108	3.493	*Int J Behav Nutr Phy*	1249
4	*International Journal of Behavioral Nutrition and Physical Activity*	71	2.296	*Prev Med*	1193
5	*Journal of Affective Disorders*	71	2.296	*BMC Public Health*	1172
6	*Preventive Medicine*	58	1.876	*Lancet*	1133
7	*BMJ Open*	56	1.811	*Am J Prev Med*	1044
8	*Frontiers in Public Health*	55	1.779	*Brit J Sport Med*	996
9	*Frontiers in Psychiatry*	31	1.003	*JAMA – J Am Med Assoc*	933
10	*Nutrients*	29	0.938	*Int J Env Res Pub He*	849

### 3.7. Analysis of highly cited articles

Among the 3092 publications, we selected the top 10 highly cited articles for analysis. The most cited article is “Lack of exercise is a major cause of chronic diseases” published in 2012,^[21]^ with 1364 citations. This article examines the impact of physical inactivity on chronic diseases. The remaining 3 articles with over 1000 citations are “Systematic review of sedentary behavior and health indicators in school-aged children and youth,” published in 2011 with 1286 citations^[22]^; “Physical activity and mental health in children and adolescents: a review of reviews,” published in 2011 with 1266 citations^[23]^; and “Systematic review of the relationships between objectively measured physical activity and health indicators in school-aged children and youth,” published in 2016 with 1156 citations.^[24]^ The article with the highest impact factor among the cited papers is “City planning and population health: a global challenge,”^[25]^ with an impact factor of 168.9, published in *Lancet*. The next highest impact factor is for “Physical activity and mental health in children and adolescents: a review of reviews,” published in the *British Journal of Sports Medicine*, with an impact factor of 18.6. The impact factors of the remaining articles are below 10. Additionally, we observed that 3 of the top 10 articles are from Canada, 3 are from England, 2 are from Australia, and 2 are from the USA, indicating that these 3 countries are core contributors to publications in this field (see Table 5).

**Table 5:** Top 10 highly cited articles.

Rank	Year	Title	First author/Country	Journal	Journal IF	Citations
1	2012	Lack of exercise is a major cause of chronic diseases	Booth, Frank W./USA	*Comprehensive Physiology*	5.8	1364
2	2011	Systematic review of sedentary behavior and health indicators in school-aged children and youth	Tremblay, Mark S./Canada	*International Journal of Behavioral Nutrition and Physical Activity*	8.7	1286
3	2011	Physical activity and mental health in children and adolescents: a review of reviews	Biddle, Stuart J. H./England	*British Journal of Sports Medicine*	18.6	1266
4	2016	Systematic review of the relationships between objectively measured physical activity and health indicators in school-aged children and youth	Poitras, Veronica Joan/Canada	*Applied Physiology Nutrition and Metabolism*	3.4	1156
5	2010	Fundamental movement skills in children and adolescents review of associated health benefits	Lubans, David R./Australia	*Sports Medicine*	9.8	903
6	2018	Physical activity and incident depression: a meta-analysis of prospective cohort studies	Schuch, Felipe B./Brazil	*American Journal of Psychiatry*	17.7	705
7	2016	City planning and population health: a global challenge	Giles-Corti, Billie./Australia	*Lancet*	168.9	608
8	2006	Adolescent physical activity and health – a systematic review	Hallal, Pedro C./Brazil	*Sports Medicine*	9.8	586
9	2004	Health-enhancing physical activity and sedentary behavior in children and adolescents	Biddle, SJH/Leics	*Journal of Sports Sciences*	3.4	574
10	2014	The effects of stress on physical activity and exercise	Stults-Kolehmainen, Matthew A./USA	*Sports Medicine*	9.8	569

### 3.8. Analysis of co-cited references

**Figure 4.**
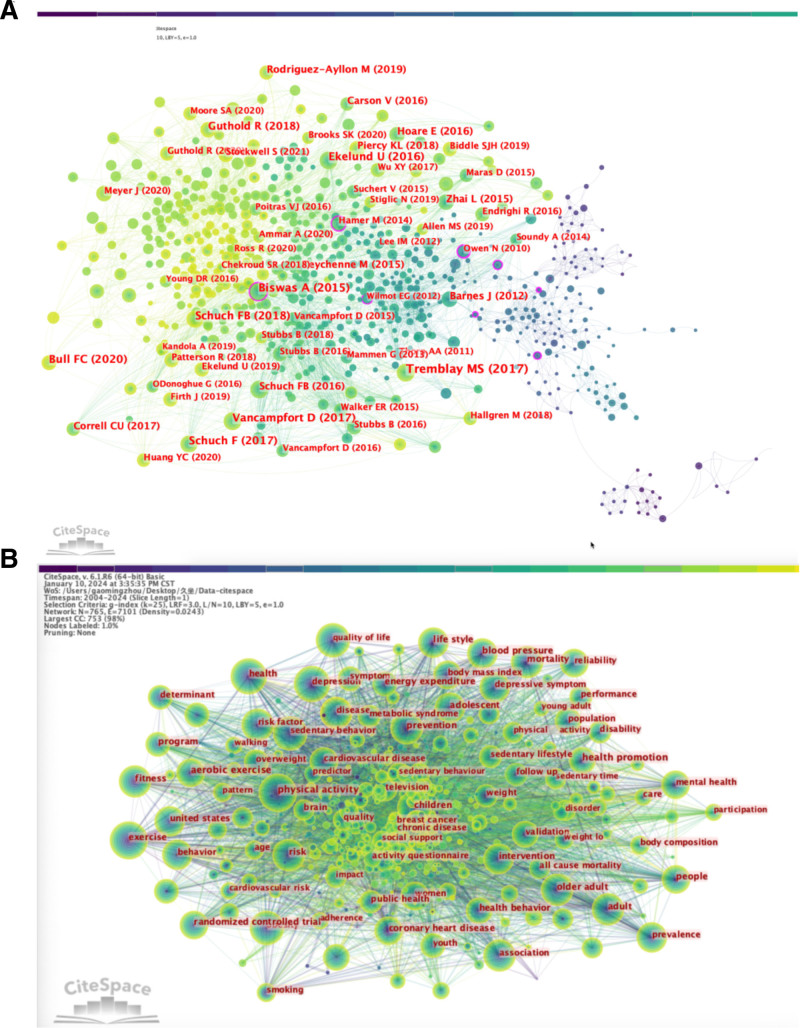
Visualization map of co-cited references (A) and co-occurrence keywords network (B).

In the literature co-citation network generated by CiteSpace, the size of nodes is proportional to the number of citations received by the papers. Larger nodes indicate higher citation counts for the corresponding papers. Among the top 10 co-cited papers, the most co-cited paper is “Sedentary Behavior Research Network (SBRN) – Terminology Consensus Project process and outcome,”^[26]^ followed by “Sedentary time and its association with risk for disease incidence, mortality, and hospitalization in adults: a systematic review and meta-analysis,”^[27]^ which is also the paper with the highest centrality. Other important publications with co-citation frequencies exceeding 100 include “Sedentary behavior and physical activity levels in people with schizophrenia, bipolar disorder and major depressive disorder: a global systematic review and meta-analysis,”^[28]^ “Physical activity and incident depression: a meta-analysis of prospective cohort studies,”^[29]^ and “Does physical activity attenuate, or even eliminate, the detrimental association of sitting time with mortality? A harmonized meta-analysis of data from more than 1 million men and women.”^[30]^ These papers hold significant positions in research in this field (see Table 6 and Fig. 4A).

**Table 6:** Top 10 co-cited reference.

Rank	Count	Centrality	Year	Author	Journal	Title
1	151	0.05	2017	Tremblay MS	*Int J Behav Nutr Phy*	Sedentary Behavior Research Network (SBRN) – Terminology Consensus Project process and outcome
2	108	0.16	2015	Biswas A	*Ann Intern Med*	Sedentary time and its association with risk for disease incidence, mortality, and hospitalization in adults a systematic review and meta-analysis
3	103	0.01	2017	Vancampfort D	*World Psychiatry*	Sedentary behavior and physical activity levels in people with schizophrenia, bipolar disorder, and major depressive disorder: a global systematic review and meta-analysis
4	102	0.03	2018	Schuch FB	*Am J Psychiat*	Physical activity and incident depression: a meta-analysis of prospective cohort studies
5	100	0.06	2016	Ekelund U	*Lancet*	Does physical activity attenuate, or even eliminate, the detrimental association of sitting time with mortality? A harmonized meta-analysis of data from more than 1 million men and women
6	93	0.01	2017	Schuch F	*J Affect Disorders*	Physical activity and sedentary behavior in people with major depressive disorder: a systematic review and meta-analysis
7	91	0.01	2020	Bull FC	*Brit J Sport Med*	World Health Organization 2020 guidelines on physical activity and sedentary behavior
8	77	0.01	2018	Guthold R	*Lancet Glob Health*	Worldwide trends in insufficient physical activity from 2001 to 2016: a pooled analysis of 358 population-based surveys with 1.9 million participants
9	74	0.04	2015	Zhai L	*Brit J Sport Med*	Sedentary behavior and the risk of depression: a meta-analysis
10	70	0.03	2016	Hoare E	*Int J Behav Nutr Phy*	The associations between sedentary behavior and mental health among adolescents: a systematic review

### 3.9. Keyword analysis

Keywords are highly condensed and summarized representations of the core content of an article, reflecting its main ideas and themes. Keywords can effectively reflect the research hotspots and trends in different periods of a field, fully embodying the articles’ themes and core research content. Excluding “sedentary behavior,” “mental health,” and “health,” the top 10 co-occurring keywords are “physical activity,” “exercise,” “association,” “depression,” “risk,” “adult,” and “quality of life.” In terms of centrality, the top 10 keywords are “physical activity” (centrality = 0.07), “aerobic exercise” (centrality = 0.07), “behavior” (centrality = 0.06), “blood pressure” (centrality = 0.06), “health promotion” (centrality = 0.05), “brain” (centrality = 0.05), “exercise” (centrality = 0.04), “adult” (centrality = 0.04), and “children” (centrality = 0.04), as shown in Table 7 and Figure 4B.

**Table 7:** Top 10 keywords from counts and centrality.

Rank	Keywords	Counts	Keywords	Centrality
1	Physical activity	1528	Physical activity	0.07
2	Sedentary behavior	969	Aerobic exercise	0.07
3	Exercise	561	Behavior	0.06
4	Association	510	Blood pressure	0.06
5	Mental health	508	Health promotion	0.05
6	Health	478	Brain	0.05
7	Depression	437	Exercise	0.04
8	Risk	408	Adult	0.04
9	Adult	332	Children	0.04
10	Quality of life	312	Older adult	0.04

**Figure 5.**
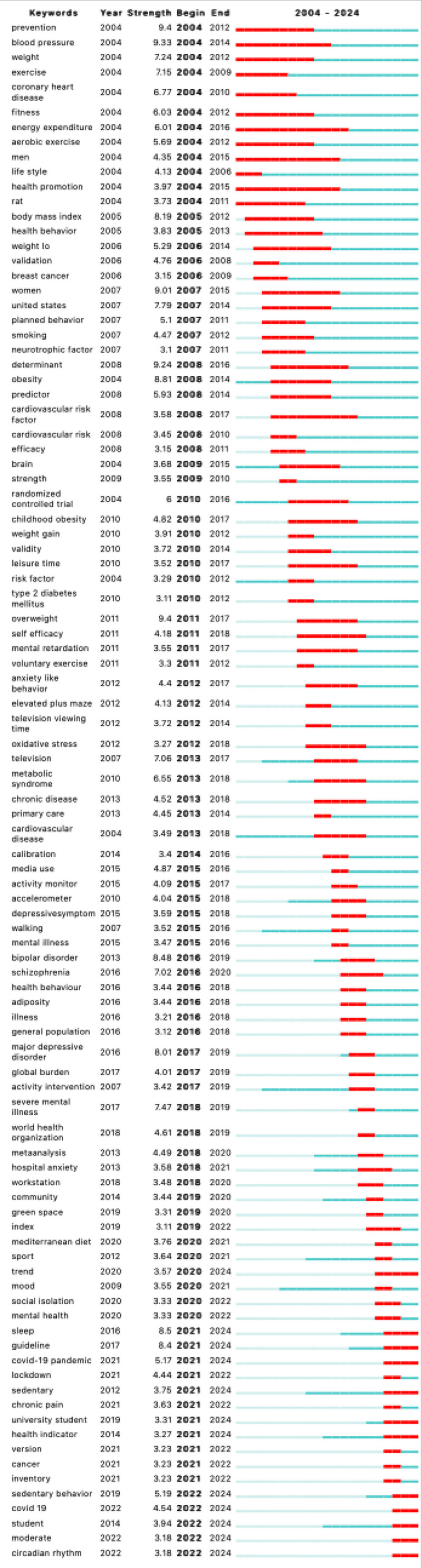
Top 96 keywords with strongest citation bursts.

Analyzing burst keywords can provide researchers with a clearer and more intuitive understanding of the hotspots and trends in the development of a field. We discovered 96 burst keywords, indicating that early research in the field, around 2004, focused on issues related to energy surplus and weight caused by prolonged sitting. From 2020 to the present, there has been increased focus on topics such as “sleep,” “COVID-19 pandemic,” “university student,” “health indicator,” and “circadian rhythm,” as shown in Figure 5.

## 4. Discussion

### 4.1. Research status

Long-term SB, frequent late nights, and lack of exercise are 3 common unhealthy habits among young people.^[31]^ The impact of SB on mental health is particularly prominent in today’s society. Therefore, we conducted a bibliometric analysis to examine the development of this field from the perspective of publications and obtained 3092 publications from 2004 to 2024. The total number of publications on “sedentary behavior and mental health” has been increasing from 2004 to 2024. The number of publications increased annually from 2004 to 2021, peaking in 2021, and has decreased annually since 2022 but still maintains a relatively high level, indicating that the field’s popularity has not waned. Moreover, the sharp increase in publications around 2021 may be significantly associated with the COVID-19 pandemic. Under the influence of the COVID-19 pandemic, sedentary time has increased, leading to an increase in psychological problems such as depression and anxiety, severely affecting the quality of life across all age groups and posing a threat to public health.^[32–35]^

Looking at the scientific collaboration in this field, the disciplinary development is driven by 102 countries/regions, 606 research institutions, and 14,881 authors. The United States and the United Kingdom contribute the most and hold core positions in the national cooperation network. Cooperation among countries in this field is relatively frequent, with the United States, Germany, Italy, England, and Spain forming the initial research core. Regarding institutional collaboration, the institution with the highest output is King’s College London, ranked in the top 10 universities in the UK (QS World Rankings 2021) and based in the heart of London. In institutional collaboration, the final node occupying a core position is UCL, which is consistently ranked as one of the top 10 universities in the world (QS World University Rankings 2010–2022). Institutions in England have made significant contributions to the study of the relationship between SB and mental health, reflecting the importance attached to research in this field by the institutions above.

Upon analyzing relevant authors, we found that among the 14,881 authors, the most prolific is Brendon Stubbs, who works at the Institute of Psychiatry, Psychology and Neuroscience, King’s College London. Brendon Stubbs also holds the highest h-index in this field. In his latest research, based on Mendelian randomization analysis, he proposed an association between SB and conditions such as schizophrenia, suggesting that PA can mitigate the risk of mental health and medication use disorders, recommending PA as a treatment modality for severe mental illness.^[36,37]^ Additionally, Davy Vancampfort from the Catholic University of Leuven has also made outstanding contributions in this field. His latest study, through a systematic review, explores the correlates of SB in people with fibromyalgia. It suggests that clinicians should consider physical and psychological barriers when reducing or interrupting SB in individuals with fibromyalgia.^[9]^

Additionally, we also examined the publication status of journals in this field. Three journals that have contributed more than 100 publications to this field are the *International Journal of Environmental Research and Public Health* (n = 226), *BMC Public Health* (n = 160), and *PLOS ONE* (n = 108). The *International Journal of Environmental Research and Public Health* and *BMC Public Health* focus on public health, with impact factors around 4, which although not high, still receive attention from researchers in this field. *PLOS ONE*, as a comprehensive journal, stands out in both publication volume and citation frequency, becoming a major journal in this field. The most cited articles published in *PLOS ONE* are “The influence of physical activity, sedentary behavior on health-related quality of life among the general population of children and adolescents: a systematic review.”^[38]^ The article suggests that “higher levels of physical activity are associated with better health-related quality of life in children and adolescents, while increased sedentary behavior is associated with lower health-related quality of life.” We also found that *Medicine & Science in Sports & Exercise*, focusing on sports medicine and exercise science, has the highest citation frequency among published articles, forming the main source of research foundation in this field.

### 4.2. Research hotspots and trends

Based on the analysis of co-cited references and keywords, the research hotspots and trends in the field of “sedentary behavior and mental health” can be summarized as follows. It was found that current researchers are focusing on topics such as “the association between sedentary behavior and physical activity with health,” “sedentary behavior and its association with depression, anxiety, and risk factors,” and “the association between sedentary behavior and obesity, cardiovascular mortality risk, and other related diseases in children and the elderly.”

Systematic reviews involving children and adults demonstrate a dose-response relationship between PA, SB, and health-related quality of life. This suggests that the higher the frequency of PA or the shorter the sedentary time, the better the health-related quality of life.^[38]^ Long-term SB is associated with a range of negative health outcomes for older adults, from poorer cardiometabolic health to impaired physical function and higher mortality risk.^[39]^ A bidirectional Mendelian randomization study indicates that PA and sedentary time have bidirectional effects on mental health outcomes.^[40]^ Further investigations, such as those conducted in Polish children and adolescents,^[41]^ are focused on explaining these associations, which essentially clarify the significant impact of SB on health.

Some researchers are also focusing on explaining the relationship between SB and current diseases, such as the impact of low-intensity PA and SB on cardiopulmonary health,^[42]^ as well as the effects of PA and SB on weight management.^[43]^ Regarding emotional impact, anxiety, and depression have always been hot topics of research. With prolonged sedentary time, the level of stress, anxiety, and depression among university students has significantly increased.^[44]^ Furthermore, SB shows a dose–response positive correlation with anxiety, depression, and suicidal behavior, highlighting the enormous harm of suicidal behavior to overall health.^[45,46]^ Research on these topics is ongoing and continues to be a sustained focus.

However, future research trends may focus on SB patterns during the COVID-19 pandemic, its impact on student sleep, and changes in related health indicators. For example, during the COVID-19 pandemic, quarantine measures have led to restricted activity, reduced PA, and increased SB among children, adolescents, and adults.^[35]^ Additionally, longitudinal Mendelian randomization studies suggest that prolonged sedentary time adversely affects COVID-19 recovery.^[46]^ Exploratory studies in 2023 further indicate that individuals with an active lifestyle and avoidance of prolonged sitting habits have a higher quality of life during the outbreak of COVID-19, suggesting that avoiding SB and adopting an active lifestyle are the best strategies in facing unpredictable pandemics.^[47]^ Moreover, a survey in Thailand revealed that 75.8% of the population experienced prolonged sitting during COVID-19, particularly those with higher education and income levels. Targeted interventions to break SB are beneficial for maintaining health.^[48]^ Additionally, numerous studies on SB and depression are currently being published, indicating that research in this area will continue for some time to come.

Certainly, with increased emphasis on health and well-being, sleep quality has received particular attention. Many studies have shown a deep connection between SB and sleep disturbances. For instance, the National Health and Nutrition Examination Survey of 22,599 participants indicated that SB is a risk factor for sleep disorders.^[8]^ Moreover, research from various perspectives indicates a relationship between SB and sleep quality. Increased PA and reduced SB are positively correlated with sleep quality.^[49]^ When considering BMI, prolonged SB (such as screen time, smartphone use, and computer usage) and higher BMI may lead to shortened sleep duration and decreased sleep efficiency in young adults in China.^[50]^ There will be more research conducted in this area in the future.

## 5. Conclusion

Over the past 2 decades, research on SB and mental health has received widespread attention, becoming a hot topic in public health. However, there has been a slight decline in publication growth after 2022. There is close collaboration among countries, institutions, and authors, with the United States, Kings College London, UCL, as well as Stubbs B and Vancampfort D, occupying core positions in this research field. Journals focusing on public health are the main sources of publications. Future research hotspots include investigating the relationship between the COVID-19 pandemic and patterns of SB, as well as the impact of SB on sleep and specific psychological disorders.

### 5.1. Relevance for clinical practice

In recent years, SB, mainly based on TV viewing time and screen time, has become increasingly common. SB mainly refers to low energy consumption behavior in which the body maintains a sitting or lying position while in a clear state. This behavior is not conducive to physical health and is considered a precursor factor affecting the body’s physical and mental health. SB can cause the occurrence and development of emotional disorders such as anxiety and depression. However, the current research status in this field is not clear. This study will help to clarify the impact of SB on mental health and provide reference for the prevention and treatment of diseases such as depression in clinical practice.

### 5.2. Limitations

This study has limitations to note. We mainly used the WOS core database, which may miss some research from other databases or gray literature. The bibliometric method, focusing on quantitative analysis, cannot fully capture qualitative details like specific contexts of how SB affects mental health. Despite these, the study provides key value. By visualizing a knowledge map, it offers a broad view of 2 decades of research trends. Identifying hotspots and future directions can guide researchers to focus on emerging areas, enhance global collaboration, and develop targeted solutions for mental health issues related to SB.

## Author contributions

**Data curation:** Yi Liu, Zhen Zhang.

**Formal analysis:** Yi Liu.

**Investigation:** Yi Liu, Sisheng Tian.

**Methodology:** Yi Liu, Sisheng Tian.

**Project administration:** Hao Zhang, Sisheng Tian.

**Resources:** Zhen Zhang, Hao Zhang, Sisheng Tian.

**Software:** Yi Liu.
